# Cocoa pulp as a novel source of plant-derived exosome-like nanovesicles: method comparison and physicochemical characterization

**DOI:** 10.3389/fbioe.2025.1729503

**Published:** 2026-01-12

**Authors:** Andrea Tapia-Aguayo, Stephanie Krüger, Ludger A. Wessjohann, María José Chica, Dariush Hinderberger, Jonas Volmer, José González-Valdez, Perla A. Ramos-Parra, Miguel Fernández-Niño

**Affiliations:** 1 Tecnologico de Monterrey, Escuela de Ingeniería y Ciencias, Monterrey, Nuevo León, Mexico; 2 Department of Bioorganic Chemistry, Leibniz-Institute of Plant Biochemistry (IPB), Halle (Saale), Germany; 3 Core Facility Microscopy, Biozentrum, Martin-Luther-University, Halle-Wittenberg, Germany; 4 CasaLuker S.A. Colombia, Bogotá, Colombia; 5 Institute of Chemistry, Physical Chemistry − Complex Self-Organizing Systems, Martin Luther University Halle-Wittenberg, Halle (Saale), Germany; 6 Department of Biotechnology, Institute of Agrochemistry and Food Technology (IATA-CSIC), Valencia, Spain

**Keywords:** cocoa residues, cryogenic grinding, high-performance homogenizing, plant-derived exosome-like nanovesicles (PENs), primary recovery operations

## Abstract

**Introduction:**

Cocoa (*Theobroma cacao* L.) processing generates substantial agricultural residues, including pod husks and mucilaginous pulp, which remain largely underutilized. In this study, we report for the first time the isolation of plant-derived exosome-like nanovesicles (PENs) from cocoa pulp, a novel biological matrix with potential applications in sustainable nanobiotechnology.

**Methods:**

Two primary cell disruption methods, high-performance homogenization and cryogenic grinding, were compared for PEN recovery, followed by ultracentrifugation-based purification. The vesicles were characterized by cryo-transmission electron microscopy (Cryo-TEM), dynamic light scattering (DLS), and bicinchoninic acid (BCA) protein assays.

**Results:**

PENs isolated via cryogenic processing exhibited more uniform size distributions (111.9 ± 2.4 nm) and spherical morphology compared to those obtained by homogenization (120.2 ± 5.5 nm). While homogenization yielded higher protein concentrations, it also resulted in greater vesicle heterogeneity. No vesicles were detected from cocoa pod husks under the tested conditions.

**Conclusion:**

These findings establish cocoa pulp as a promising and sustainable source of PENs and demonstrate how primary recovery methods critically influence vesicle quality. This methodological contribution lays the groundwork for future studies on PEN bioactivity, cargo profiling, and functional applications.

## 1 Introduction

Every year, over 3.5 million tons of cocoa residues are generated globally by the chocolate and other cocoa-derived product industries (i.e., paste, butter, powder, and liquor). Each mature cocoa fruit contains between 20 and 40 cocoa beans, surrounded by a mucilaginous pulp that accounts for approximately 20% of the fruit’s weight. In contrast, the fruit’s peel or shell (commonly known as pod husk) makes up to 80% of the fruit’s weight. Considering a global annual production of nearly 5 million tons of cocoa, the pulp and pod husk together constitute the main agro-industrial residues of cocoa processing, representing a significant management challenge for cocoa-producing countries (de Souza et al., 2018; Swiss Platform for Sustainable Cocoa, 2024; Sánchez et al., 2024; Vásquez et al., 2019).

Recently, the circular economy has gained prominence as a strategy to mitigate environmental degradation by promoting the restorative use of manufacturing by-products that are not necessarily considered waste (Ricciardi et al., 2020; Arpit Singh et al., 2022). Given their rich content of nutritional and bioactive compounds, such as fibers, phenols, vitamins, carbohydrates, fats, minerals, aromatic compounds, and other molecules, cocoa residues present a valuable opportunity for resource reutilization. Their abundance, renewability, and low cost further enhance their economic potential while reducing the environmental impact of the cocoa industry (Vásquez et al., 2019; Arpit Singh et al., 2022; Mora-Garrido et al., 2022). Globally, many companies are exploring their use in products such as antimicrobial and anti-glucosyltransferase extracts, dietary fiber, cocoa-based beverages, and the extraction of theobromine and caffeine (Vásquez et al., 2019). Specifically, cocoa pulp is rich in sugars and minerals, and lacks alkaloids or other toxic substances, whereas cocoa pod husk is rich in lignin, non-starch polysaccharides (e.g., cellulose, hemicellulose, and pectin), terpenoids (e.g., chrysophanol), phenolic acids (e.g., protocatechuic, salicylic), carboxylic acids (e.g., citric, tartaric), and free amino acids (e.g., glutamine, asparagine, serine, and lysine) (Sánchez et al., 2024; Soares and Oliveira, 2022).

Besides their importance in the extraction of bioactive compounds, agro-industrial residues offer a compelling opportunity to produce biological nanoparticles. Among these, plant-derived exosome-like nanovesicles (PENs) have emerged as promising candidates for biomarker discovery and drug delivery applications (Ayala-Mar et al., 2019a; Ayala-Mar et al., 2019b). Once considered simple waste disposal vehicles, PENs are now recognised for their crucial roles in defense, intercellular transport, growth, and plant–microbe interaction (Rutter and Innes, 2017; Cui et al., 2020). Despite persistent challenges in their isolation and the relatively early stage of PEN research, these nanovesicles have demonstrated the ability to modulate immune responses, transport bioactive molecules, and facilitate intercellular communication, opening new avenues in plant biotechnology and therapeutic innovation (Cui et al., 2020).

In this study, we investigated the presence of exosome-like nanovesicles from the pulp and pod husks of *Theobroma cacao* (cocoa). We successfully purified and characterized PENs from cocoa pulp, highlighting the potential of this industrial residue as a novel source of these vesicles. To achieve this, high-performance homogenization (i.e., Ultra-Turrax homogenizer) and cryogenic grinding (i.e., liquid nitrogen freezing) were evaluated and compared as primary cell disruption methods prior to purification via ultracentrifugation. Our findings revealed significant differences in the physicochemical properties of the PENs obtained using the two treatments and support the strategic use of cocoa pulp as a sustainable input for nanobiotechnological applications.

## 2 Materials and methods

### 2.1 Cocoa pulp and pod husk procurement

Cocoa pulp and pod husks were donated by Casa Luker S.A., a leading producer of fine aroma cocoa in Colombia. The pulp consisted of a composite mix derived from several premium cocoa varieties, including LUKER40, CCN 51, Colombia Selection, TCS6, FEAR5, CAUCASIA 39, L61, TSH 565, and ICS39. Pod husks from the same fine cocoa clones were also provided. All samples were stored at −80 °C immediately upon arrival and maintained under these conditions until further processing.

### 2.2 Primary recovery of cocoa-derived PENs

5 g of cocoa pulp or pod husk were processed using two distinct cell disruption methods to release PENs: high-performance homogenization and cryogenic grinding. For the homogenization treatment, samples were processed using a T25 Ultra-Turrax homogenizer (IKA Labortechnik, Germany). Each 5 g sample was placed in a sterile tube containing 5 mL of phosphate-buffered saline (PBS, pH 7.4) and homogenized for 1 min at 3000 rpm (the lowest homogenizer speed) in an ice bath. The homogenate volume was then adjusted to 15 mL with the same PBS solution. For the cryogenic grinding treatment, 5 g of cocoa pulp or pod husk was placed in a mortar and immersed in liquid nitrogen until fully frozen. The frozen sample was macerated with a pestle until a homogeneous paste was obtained, which was then transferred into sterile tubes using PBS, with the final volume adjusted to 15 mL. All processed samples were stored at 4 °C until further use.

### 2.3 Cocoa-derived PEN purification

PEN purification was performed following the procedure outlined by Perut et al., (2021), with some modifications. The vesicles were isolated via ultracentrifugation using an Avanti JXN-30 centrifuge (Beckman Coulter, USA) equipped with a JS-25.15 rotor (Beckman Coulter, USA). Initially, the samples were centrifuged at 3,500 x g for 30 min at 4 °C using ultra-clear centrifuge tubes (Beckman Coulter, USA). The supernatant was transferred to a new tube and subjected to differential centrifugation at 3,500 x g for another 30 min at 4 °C, followed by ultracentrifugation at 16,500 x g for 45 min at 4 °C. The resulting supernatant was then filtered through a 0.45 µm syringe filter before a second ultracentrifugation at 110,000 x g for 1.5 h at 4 °C. The pellet was washed with sterile PBS (pH 7.4) and centrifuged again at 110,000 x g for 1.5 h at 4 °C. Finally, the resulting pellet was resuspended in 500 µL of sterile PBS and stored at −80 °C until further use.

### 2.4 PEN characterization

#### 2.4.1 Morphology determination

PEN morphology was assessed using cryo-transmission electron microscopy (Cryo-TEM). Imaging was performed on a Carl Zeiss Libra 120 microscope (Carl Zeiss, Germany) operating at 120 kV, equipped with a TSR Dual Speed 2k × 2k CCD camera (Tröndle Restlichtverstärkersysteme, Germany). Sample preparation was conducted as follows: copper mesh 200 C-Flat 4/2 2-Cu T grids (Electron Microscopy Sciences (EMS), USA) were glow-discharged in air for 25 s immediately before use. A volume of 3 µL/side of the sample was applied to each grid, blotted for 10 s, and rapidly plunged into liquid ethane. The plunger was set to 19 °C with 80% relative humidity.

#### 2.4.2 Particle size and surface charge/zeta potential determination based on dynamic light scattering (DLS)

DLS measurements were conducted using a Litesizer 500 instrument (Anton Paar GmbH, Austria). PEN samples derived from cocoa pulp and cocoa pod husk were diluted 1:10 in sterile phosphate-buffered saline (PBS, pH 7.4). A 60 µL aliquot of each sample was transferred into a quartz cuvette (Hellma Analytics, Germany) for analysis. Measurements were performed at 25 °C, using six runs of 10 s each and an equilibration time of 1 min with three scattering angles. Automatic focus was enabled. Data were processed using the general analysis model, with the refractive index of PENs set to 1.3700 and PBS sterile solution as the solvent (refractive index n_PBS_ = 1.3318, viscosity h = 0.0009041 Pa.s). Hydrodynamic diameters and zeta potential values were calculated using Kalliope Software v2.22.0 (Anton Paar, Austria).

#### 2.4.3 Total protein quantification

Nanoparticle concentration was expressed in terms of total protein content, quantified using the bicinchoninic acid (BCA) protein assay kit (Thermo Scientific, Rockford, IL), following the protocol adapted from Mahdipour (2022), which is specifically optimized for plant-derived extracellular vesicles. A bovine serum albumin (BSA) calibration curve was prepared within the range of 0–2000 μg/mL, yielding the following equation: y = 0.0007x + 0.0265 (*R* ^2^ = 0.9992). For the assay, 9 µL of each PEN sample were mixed with 260 µL of BCA working reagent in a 96-well clear polystyrene microplate (Corning, USA). The plate was incubated at 37 °C for 30 min, and absorbance was measured at 562 nm using a microplate reader. All measurements were performed at least by triplicate to ensure reproducibility.

### 2.5 Statistical analysis

All results are presented as mean ± standard error (SE) from at least three independent replicates. Statistical analyses (i.e., Welch’s t-test) were conducted using GraphPad Prism software, version 9.0.1 (128) (GraphPad Software, USA).

## 3 Results and discussion

To the best of our knowledge, this is the first report describing the isolation and characterization of PENs from cocoa pulp. Cocoa pulp and cocoa pod husk samples were subjected to two different mechanical disruption techniques—high-performance homogenization using an Ultra-Turrax and cryogenic grinding with liquid nitrogen—to evaluate their effectiveness as primary recovery methods for PEN isolation. The resulting extracts were processed using a standardized ultracentrifugation-based purification protocol, as detailed in the and previously described by Perut et al. (2021). Notably, once the PENs were isolated, their morphology and protein content differed significantly depending on the initial disruption method, suggesting that the primary recovery step has a substantial impact on the physicochemical properties of the resulting nanovesicles. These differences are further detailed below.

As with other high-value biotechnological products, efficient cell disruption is a key requirement for primary recovery in PEN isolation. However, in this case, the disruption method not only affects the efficiency of nanovesicle release, but also plays a crucial role in preserving their physicochemical integrity. Despite the growing interest in PENs, there is still limited information on how different primary recovery procedures influence both yield and vesicle quality, regardless of the plant source. Notably, most reported protocols transform plant materials into a liquid form, such as fruit juices, prior to PEN isolation. Some studies have even described highly variable and non-standardized approaches, for instance, using manual extrusion through filter paper (Ito et al., 2021), or household blenders for tissue homogenization (Kim et al., 2022; Yang et al., 2024). These inconsistencies highlight the need for more systematic evaluations of how initial sample processing impacts the structural and functional characteristics of PENs.

The wide range of primary recovery methods reported for PEN isolation reflects both the diversity of plant and vegetable sources from which these vesicles can be obtained, and the increasing interest in revalorizing complex agro-industrial waste streams. Additionally, many published protocols offer only vague guidance on this step, often referring to it simply as “grinding” without further standardization or optimization (Li et al., 2024). As a result, primary recovery operations remain largely overlooked, despite their crucial influence on nanovesicle yield and quality. As PEN-based applications move closer to commercialization, a more systematic understanding and optimization of these initial processing steps will become essential to ensure reproducibility, scalability, and product integrity.

With this in mind, we evaluated the efficiency of PEN isolation from cocoa pulp and pod husk tissues, using two fundamentally different mechanical disruption methods. To understand the impact of each approach, we compared the resulting nanovesicles in terms of their morphology, total protein content, size distribution, and surface charge. These comparative analyses provide initial insights into how the chosen disruption technique influences the physicochemical characteristics of the extracted PENs.

**FIGURE 1:**
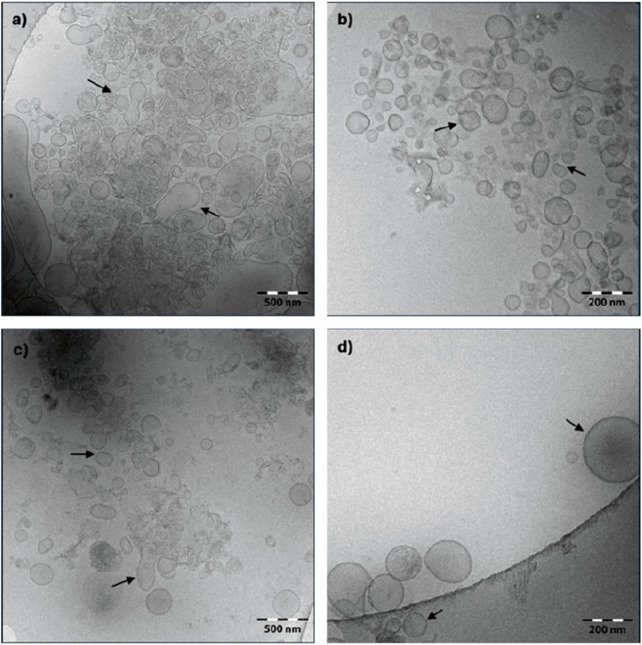
Morphological characterization of exosome-like nanovesicles derived from cacao pulp by cryogenic transmission electron microscopy (Cryo-TEM). Images were acquired using a Carl Zeiss Libra 120 microscope at 120 kV equipped with a TSR Dual Speed 2k × 2k CCD camera. **(a,b)** Show vesicles isolated via high-performance homogenization at different magnifications: **(a)** low magnification (scale bar: 500 nm) and **(b)** high magnification (scale bar: 200 nm). **(c,d)** show vesicles obtained through cryogenic grinding at: **(c)** low magnification (scale bar: 500 nm) and **(d)** high magnification (scale bar: 200 nm). The range of magnifications enables both general morphological assessment and detailed structural visualization. Cocoa pulp exosome-like nanovesicles are indicated by arrows.

Cryo-TEM analysis of cocoa pulp-derived PENs revealed that nanovesicles from both primary recovery treatments exhibited generally spherical morphologies (Figure 1). However, notable differences were observed between the two methods. PENs obtained via high-performance homogenization displayed irregular membrane contours and less uniform shapes (Figures 1a,b), whereas those recovered through cryogenic grinding showed more consistent, well-defined, and in many cases, nearly perfect spherical structures (Figures 1c,d). In terms of size, vesicles derived from homogenization had diameters ranging from 50 to 250 nm, while those isolated following cryogenic grinding ranged from 50 to 200 nm, indicating a slightly narrower and more homogeneous size distribution in the latter group.

**FIGURE 2:**
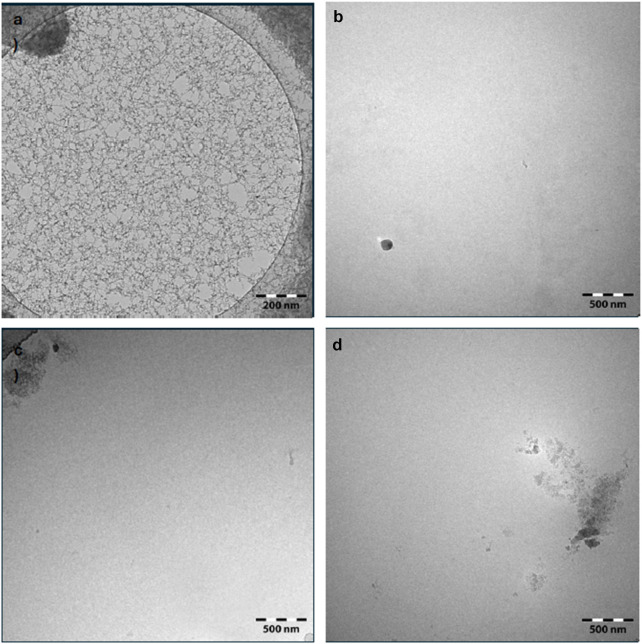
Morphological characterization of nanostructures derived from cocoa pod husk by cryogenic transmission electron microscopy (Cryo-TEM). Imaging was performed using a Carl Zeiss Libra 120 microscope at 120 kV equipped with a TSR Dual Speed 2k × 2k CCD camera. **(a,b)** show samples processed by high-performance homogenization, acquired at different magnifications: **(a)** low magnification (scale bar: 200 nm) and **(b)** high magnification (scale bar: 500 nm). **(c,d)** depict samples processed by cryogenic grinding at high magnification (scale bar: 500 nm). Cocoa pod husk cellulose-based nanofibers were observed.

In contrast, no detectable nanovesicles were recovered from cocoa pod husk samples, regardless of the disruption method used (Figure 2). This absence is likely attributed to the tissue’s high content of structural polysaccharides such as cellulose, hemicellulose, lignin, and pectins, which may hinder effective cell disruption and vesicle formation or release. Notably, Cryo-TEM imaging revealed the presence of cellulose nanofibers, further supporting the structural rigidity and resistance of this matrix to vesicle liberation (Figures 2a,b). Despite this limitation, identifying viable strategies to repurpose cocoa pod husk remains important, as this by-product is commonly discarded as residual biomass, contributing to environmental issues, including the proliferation of pathogenic microorganisms (Holguín Posso et al., 2024).

**FIGURE 3:**
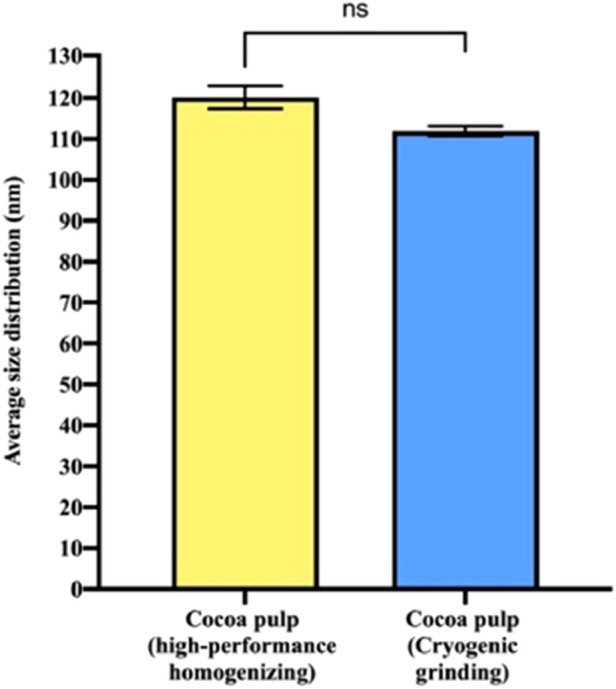
Dynamic light scattering (DLS) analysis of exosome-like nanovesicles (PENs) derived from cocoa pulp after primary recovery by two different processing methods. Samples obtained via high-performance homogenization exhibited an average particle size of 120.15 ± 5.52 nm, while those obtained through cryogenic grinding showed an average size of 111.92 ± 2.35 nm. Bars represent mean ± standard error (n = 3). No statistically significant difference was observed between the two groups (ns, Welch’s t-test).

In line with the morphological observations, dynamic light scattering (DLS) analysis showed that PENs derived from both primary recovery methods exhibited comparable average diameters. Specifically, vesicles obtained through high-performance homogenization had an average size of 120.15 ± 5.52 nm with a polydispersity index (PDI) of 0.18 ± 0.048, while those recovered via cryogenic grinding measured 111.92 ± 2.35 nm with a PDI of 0.23 ± 0.019, with no statistically significant differences between the two groups (Figure 3). Importantly, both PEN samples exhibited PDI values within the 0.1–0.25 range, which is characteristic of narrow and acceptable size distributions for nanovesicle preparations (Hoseini et al., 2023). However, the size distribution profiles revealed notable distinctions in vesicle population heterogeneity depending on the disruption technique employed (Table 1). High-performance homogenization yielded four distinct size populations, indicating a broader and more heterogeneous vesicle distribution. This finding aligns with Cryo-TEM images, which also revealed greater morphological variability in these samples. In contrast, cryogenic grinding produced a more uniform vesicle population, with only two predominant size groups, corroborating the narrower and more consistent morphology observed via TEM.

**TABLE 1:** Size distribution of cocoa-derived exosome-like nanovesicles (PENs) obtained through high-performance homogenization and cryogenic grinding, as assessed by dynamic light scattering (DLS).

DLS size populations	High-performance homogenizing		Cryogenic grinding	
	Size (nm)	Relative percentage (%)	Size (nm)	Relative percentage (%)
1	26.47 ± 3.91	20.19	98.43 ± 5.79	76.87
2	80.54 ± 1.77	19.78	142.30 ± 9.86	23.13
3	115.80 ± 5.00	31.30	—	—
4	167.95 ± 1.65	28.73	—	—

The table summarizes the average hydrodynamic diameter (mean ± standard deviation) and relative percentage of particles within each size distribution group for both processing methods. Multiple size populations were detected in samples processed by high-performance homogenization, while cryogenic grinding predominantly yielded one major population. ‘—’ indicates absence of a corresponding size group in the distribution profile.

These observed differences can be attributed to the distinct mechanical principles underlying each grinding method. High-performance homogenization applies intense shear and pressure forces that tend to disrupt cellular structures in a less controlled manner, resulting in heterogeneous fragmentation and the formation of vesicles with variable sizes and irregular morphologies. In contrast, cryogenic grinding in the presence of liquid nitrogen enables rapid flash freezing, which facilitates more uniform and brittle fracture of the tissue. This likely contributes to a more controlled rupture of cellular components, better preservation of vesicle structure, and a narrower size distribution. Despite these differences, the similar mean diameters and zeta potential values observed between the two methods indicate that both approaches can yield PENs with broadly comparable physicochemical properties. Nevertheless, the choice of grinding technique significantly influences the homogeneity of the resulting vesicle population.

The Cryo-TEM and DLS results obtained in this study were consistent with each other (Filippov et al., 2023), as the spherical morphology observed by TEM corresponded well with the hydrodynamic diameters measured by DLS and in agreement with previously reported size ranges for PENs from different plant species. For instance, Ye et al., (2024) reported PENs from *Momordica charantia L.* with an average diameter of approximately 155 nm. Zhao et al., (2023) described *Allium sativum* (garlic) PENs ranging from 44 to 396 nm. Similarly, Cao et al., (2019) and Han et al., (2022) reported *Panax ginseng* (ginseng) PENs with diameters between 200 and 345 nm. Yang et al., (2021) also found *M. charantia L.* (bitter melon) PENs within the 100–150 nm range. In addition, Potestà et al., (2020) identified *Moringa oleifera Lam.* (moringa) nanovesicles with sizes between 240 and 500 nm, while *Dendropanax morbiferus*-derived vesicles were reported to range from 100 to 200 nm (Kimin et al., 2020). These comparisons further support the validity of the size profiles observed in our cocoa pulp-derived PENs and underscore the diversity in PEN size across different plant species.

Overall, the observed size differences can be attributed to the distinct mechanical and physicochemical effects exerted by each cell disruption method. High-performance homogenization relies on intense shear forces to effectively break down cellular structures, often resulting in higher vesicle release (IKA, 2023). However, the high-energy impact generated by acceleration forces, turbulence, and shear and thrust stresses can lead to heterogeneous fragmentation, producing vesicles with variable sizes and potential structural alterations (Elbendari and Ibrahim, 2025). Also, it cannot be excluded that the process itself is generating vesicles *de novo* from membranes. In contrast, cryogenic grinding exposes the sample to rapid freezing, which better preserves vesicle integrity while minimizing mechanical stress. This likely accounts for the more uniform and spherical morphology observed in these vesicles, as the freezing process reduces uncontrolled membrane deformation (Ebrahimi et al., 2022). It is also important to consider that the specific biochemical composition and molecular characteristics of the vesicle surface may influence vesicle–vesicle interactions, thereby affecting both size distribution and total protein content.

**FIGURE 4:**
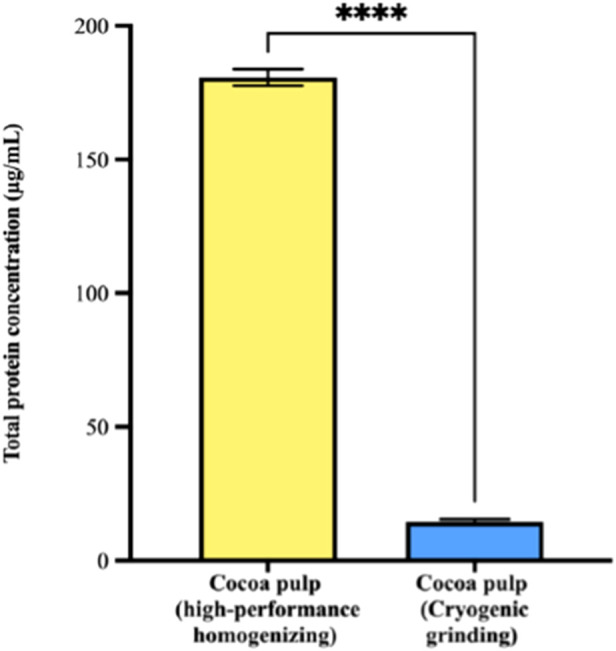
Total protein concentration of exosome-like nanovesicles (PENs) isolated from cocoa pulp following primary recovery via high-performance homogenization or cryogenic grinding. Quantification was performed using the bicinchoninic acid (BCA) assay. Cocoa pulp-derived PENs isolated through high-performance homogenization exhibited a markedly higher protein concentration (180.71 ± 7.69 μg/mL) compared to those obtained via cryogenic grinding (14.57 ± 2.19 μg/mL). Bars represent mean protein concentration ±standard error (****p < 0.0001, n = 3), indicating a highly significant difference between the two methods.

Total protein concentration, as determined by the BCA assay, revealed a significant difference between the two recovery methods. Cocoa pulp-derived PENs isolated through high-performance homogenization exhibited a markedly higher protein concentration (180.71 ± 7.69 μg/mL) compared to those obtained via cryogenic grinding (14.57 ± 2.19 μg/mL) (Figure 4). This substantial difference was statistically significant (p < 0.0001), clearly demonstrating the strong influence of the chosen primary recovery technique on the protein content of the resulting nanovesicles.

The observed differences in protein concentration can be attributed to the distinct mechanical effects of each technique. Homogenization generates high shear forces, which may lead to more efficient cell disruption and, consequently, greater release or even new formation of vesicles and their associated proteins. In contrast, cryogenic grinding, while better preserving vesicle integrity, may limit the release of vesicles, thus resulting in lower protein concentrations. This finding is particularly relevant, since the protein content of PENs is directly related to their concentration, functionality and potential applications in biotechnology and biomedicine. Consequently, our findings underscore the importance of selecting an appropriate sample pretreatment method, tailored to the specific requirements of the intended application of the isolated vesicles.

**FIGURE 5:**
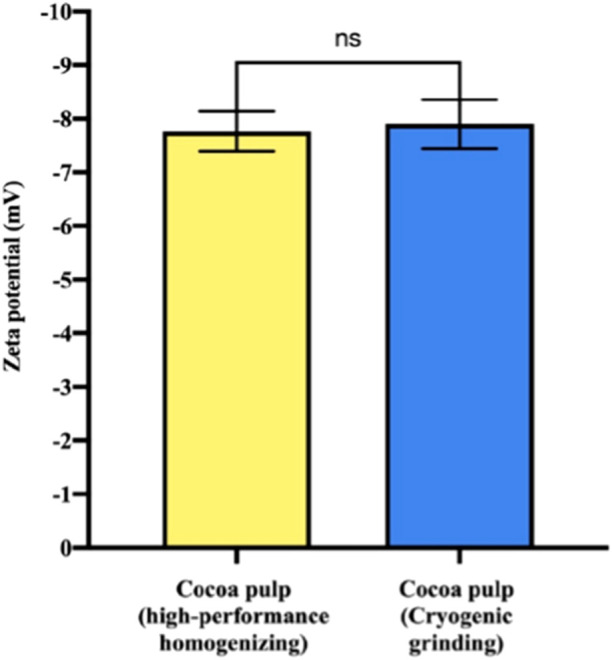
Dynamic light scattering (DLS) analysis of exosome-like nanovesicles derived from cocoa pulp after primary recovery by two different processing methods. The zeta potential of samples obtained via high-performance homogenization was −7.8 mV, while those obtained through cryogenic grinding had a zeta potential of −7.9 mV. Bars represent mean ± standard error (n = 3. No statistically significant difference was observed between the two groups (ns, Welch’s t-test).

Zeta potential analysis of cocoa pulp-derived PENs revealed similar values for both processing methods, with vesicles obtained through high-performance homogenization showing a zeta potential of −7.8 ± 0.37 mV and those obtained via cryogenic grinding exhibiting −7.9 ± 0.46 mV (Figure 5). These values fall within the expected range for plant-derived extracellular vesicles (EVs), which typically present slightly negative to moderately negative surface charges (Paulaitis et al., 2018). The narrow dispersion observed in our measurements further indicates good reproducibility and comparable colloidal behaviour between both preparations. For Plant-derived exosome-like nanovesicles, zeta potential generally range between neutral to −50 mV(32). The negative zeta potential is primarily attributed to the presence of glycosylated proteins on the lipid bilayer surface of the vesicles, which carry a negative charge. This surface charge plays a crucial role in the stability of nanovesicles and influences their behaviour, as it contributes to electrostatic repulsion between vesicles, preventing aggregation and enhancing their dispersion (Midekessa et al., 2020).

DLS analysis, including size distribution and zeta potential, revealed no statistically significant differences between vesicles obtained by the two methods. This suggests that, despite the observed differences in yield and morphology, and as indicated by protein quantification and microscopy analysis, the size distribution and surface charge of the vesicles were comparable for both pretreatment methods. The lack of statistical significance implies that the mechanical disruption method does not have a major impact on vesicle size or surface charge, at least under the conditions tested. This finding is noteworthy, as it indicates that once the vesicles are released, they maintain their biophysical properties regardless of the sample processing method. Overall, this is advantageous, as it suggests that both pretreatment techniques produce PENs with similar colloidal stability and potential biological functionality. Given that both methods yield vesicles with comparable physicochemical characteristics, researchers could choose the most appropriate pretreatment method based on factors such as processing efficiency, cost-effectiveness or protein content.

## 4 Conclusion

In this study, we compared two primary recovery methods for isolating PENs from the two major residues of the cocoa industry: cocoa pulp and cocoa pod husk. These methods involved ultracentrifugation using an ultraturrax-type homogenizer for high-performance homogenizing and cryogenic grinding with liquid nitrogen. The results revealed significant differences in the PENs isolated from cocoa pulp depending on the treatment method. PENs obtained through cryogenic grinding with liquid nitrogen treatment exhibited greater homogeneity in both shape and size compared to those obtained via high-performance homogenizing. This suggests that cryogenic grinding with liquid nitrogen may be more effective in producing uniform populations of PENs with lower (for some applications undesired) native protein content, making it a promising method for their isolation. It is important to note, however, that no exosome-like nanovesicles were found in the cocoa pod husk samples treated by either method. This suggests that the cocoa pod husk matrix may not be suitable for PENs isolation under the conditions tested, possibly due to the unique composition and structural characteristics of this plant material.

As the first report on PENs isolated from cocoa pulp, it presents new opportunities for exploring these extracellular vesicles. The promising results from cryogenic grinding highlight the potential of this method as a tool for efficiently isolating less disturbed PENs from plant by-products. However, further research is needed to optimize the conditions and fully explore the potential of this technique for various applications. Future studies should also consider other factors that might influence the properties of the isolated vesicles over time, such as storage conditions, buffer composition, and the inclusion of additional purification steps such as extra filtration steps or size-exclusion chromatography. Long-term stability assessments and functional differences between vesicles isolated by different methods will provide deeper insights into their suitability for specific applications. Incorporating metabolomic, lipidomic and proteomic analyses to characterize the chemical composition of these PENs will further enhance our understanding of their composition and potential. Overall, these findings expand our knowledge of plant-derived exosome-like nanovesicles and provide a foundation for potential applications in biotechnology and for future studies in this promising field.

## Data Availability

The raw data supporting the conclusions of this article will be made available by the authors, without undue reservation.
